# Usefulness and limitations of E-cadherin and β-catenin in the classification of breast carcinomas in situ with mixed pattern

**DOI:** 10.1186/1746-1596-8-114

**Published:** 2013-07-09

**Authors:** Douglas S Gomes, Simone S Porto, Rafael M Rocha, Helenice Gobbi

**Affiliations:** 1 Breast Pathology Laboratory, School of Medicine, Federal University of Minas Gerais (UFMG), Av. Professor Alfredo Balena, 190, Belo Horizonte, Minas Gerais, 30130-100, Brazil; 2 Laboratory of Investigative Pathology, A.C. Camargo Hospital, São Paulo-SP, Brazil

**Keywords:** E-cadherin, β-catenin, Breast cancer, Lobular neoplasia, Ductal carcinoma *in situ*, Immunohistochemistry

## Abstract

**Background:**

The distinction between lobular neoplasia of the breast and ductal carcinoma *in situ* has important therapeutic implications. In some cases, it is very difficult to determine whether the morphology of the lesion is ductal or lobular. The aim of this study was to evaluate the value of E-cadherin and β-catenin expression through the immunophenotypical characterization of carcinoma *in situ* with mixed pattern (CISM).

**Methods:**

A total of 25 cases of CISM were analyzed considering cytology/mixed architecture (ductal and lobular), nuclear pleomorphism, loss of cell cohesion, and presence of comedonecrosis. The immunophenotype pattern was considered E-cadherin positive and β-catenin positive, or negative.

**Results:**

Nineteen (76%) cases presented a mixed cytology and / or architectural pattern, two (8%) presented nuclear pleomorphism, two (8%) presented mixed cytology and nuclear pleomorphism, and two (8%) presented comedonecrosis and nuclear pleomorphism. A complete positivity for E-cadherin and β-catenin was observed in 11 cases (44%). In one case, the lesion was negative for both markers and showed nuclear pleomorphis. Thirteen lesions showed negative staining in areas of lobular cytology and positive staining in cells presenting the ductal pattern.

**Conclusions:**

The expression of E-cadherin and β-catenin, combined with cytological and architectural analysis, may highlight different immunophenotypes and improve classification of CISM.

**Virtual Slides:**

The virtual slide(s) for this article can be found here: http://www.diagnosticpathology.diagnomx.eu/vs/1693384202970681

## Abstract (pt)

**Additional non-English language abstract - Portuguese:**

**Introdução**

A distinção entre neoplasia lobular e carcinoma ductal *in situ* tem importantes implicações terapêuticas. No entanto, em alguns casos, é muito difícil determinar se a morfologia da lesão é ductal ou lobular. O objetivo deste estudo foi avaliar a expressão de E-caderina e β-catenina na caracterização imunofenotípica dos carcinomas *in situ* de padrão misto (CISM).

**Métodos**

Um total de vinte e cinco casos de CISM foram analisados considerando a citologia/arquitetura mista (ductal e lobular), pleomorfismo nuclear e presença de comedonecrose. A expressão imuno-histoquímica foi considerada positiva para E-caderina e β-catenina, ou negativa.

**Resultados**

Dezenove (76%) casos apresentavam somente citologia e/ou padrão arquitetural misto (ductal e lobular), dois casos (8%) apresentaram somente pleomorfismo nuclear, dois casos (8%) apresentavam citologia mista e pleomorfismo nuclear, e dois casos (8%) tinham comedonecrose e pleomorfismo nuclear. Uma positividade completa para E-caderina e β-catenina foi observada em 11 casos (44%). Em um caso a lesão foi negativa para ambos marcadores e apresentava pleomorfismo nuclear e comedonecrose. Em 13 lesões o imunofenótipo foi negativo em áreas lobulares e positivo em áreas ductais.

**Conclusão**

A caracterização imunofenotípica com E-caderina e β-catenina, combinada com a análise citológica e arquitetural, pode destacar diferentes imunofenótipos e auxiliar na classificação dos CISM.

## Background

*In situ* breast carcinomas are classified, according to their morphology, as ductal carcinoma *in situ* (DCIS) or lobular neoplasia (LN), which includes lobular carcinoma *in situ* (LCIS) and atypical lobular hyperplasia (ALH). According to the 2012 WHO classification of tumors of the breast, classic LCIS is diagnosed when more than half of the acini of a lobular unit are distended and distorted by a dyshesive proliferation of cells with small, uniform nuclei. Lesser involvement by the characteristic cells is diagnosed as ALH. Lesions that show marked nuclear pleomorphism, with or without apocrine features and comedonecrosis are referred as pleomorphic LCIS (PLCIS) [1].

In some cases, the diagnostic criteria based on the morphology of LN is not clear, leading to mistaken diagnosis of intraductal proliferative lesions. The main differential diagnoses of lobular neoplasia are: LN with solid low-grade DCIS, PLCIS and high-grade DCIS. Some *in situ* carcinomas present unusual cytological and / or architectural features, making it difficult to determine whether the proliferation is lobular or ductal. This group has been called carcinomas *in situ* with a mixed or indeterminate pattern (CISM) [2,3].

The differential diagnosis of the CISM carries some important implications. Patients with LN are usually clinically monitored and can be offered tamoxifen as a prophylactic therapy to prevent the development of invasive carcinoma [4,5]. On other hand, patients with DCIS should be treated by surgical removal of the lesion, with clear margins followed by radiotherapy, or mastectomy [6]. When diagnosed by core biopsy, DCIS should be treated with complete excision of the lesion. However, the clinical significance and therapeutic implications of finding LN in core biopsy specimens are still controversial [7,8].

The diagnosis of CISM is extremely rare and studies assessing the differential diagnosis of these lesions are scarce and include only a few patients. The largest series reported between 12 and 28 cases [9,10]. Previous studies by our group identified 0.08% of CISM among breast biopsies performed in our general hospital [11]. Although rare, when analyzed under light microscope, the CISM lesions are difficult to diagnose and there is lack of epidemiological data linked to their biological behavior.

A great progress in the diagnosis of these lesions came with the observation that almost all cases of LN and invasive lobular carcinoma (ILC) lose the immunohistochemistry (IHC) signal for E-cadherin and β-catenin expression in the cytoplasm membrane, whereas the expression of these proteins is maintained in both *in situ* and invasive ductal carcinomas [3,12,13]. The cadherins comprise a large number of adhesion molecules localized in the intercellular junctions, keeping cells connected through homophilic protein-protein interactions. The observation that cadherins play an important role in the establishment of the epithelial phenotype, cell migration, cell differentiation, and tumor dissemination has stimulated great interest in this family of adhesion molecules. Among them is the Human Epithelial Cadherin (E-cadherin), a calcium-dependent transmembrane glycoprotein directly involved in the process of cell adhesion [14]. The α, β, p, and γ catenins play important roles in intercellular signal transduction. The β-catenin, specifically, binds to the cytoplasmatic portion of the E-cadherin and to the structure of actin microfilaments of the cytoskeleton as well, being involved in cell adhesion [15,16]. The E-cadherin gene mutation is the major mechanism responsible for its inactivation in cancer cells and is associated with other carcinomas, such as hepatocellular carcinoma, diffuse-type gastric cancer, thyroid and colorectal cancer [16,17]. Another route resulting in inactivation of E-cadherin is attributed to dysfunctional promoter activity or DNA methylation at the promoter region [17,18].

The aim of this study was to evaluate the expression of E-cadherin and β-catenin for the immunophenotypical characterization of carcinomas *in situ* with mixed pattern, and identify potential morphological patterns that could assist in the diagnosis of the different types of CISM lesions.

## Methods

This is a retrospective, descriptive study that analyzed 25 cases of breast biopsies (wide local excision or mastectomy) performed between 1999 and 2011. The study analyzed the histopathological reports and slides available at the Laboratory of Breast Pathology at the School of Medicine at the Federal University of Minas Gerais (UFMG), Brazil. We selected one or more slides stained with hematoxylin and eosin (HE) and which were representative of each diagnosis of *in situ* carcinoma with mixed pattern. The slides were analyzed simultaneously by two of the authors (HG and DSG) using a dual-view microscope and classified according to the standard morphological patterns and immunohistochemical expression of E-cadherin and β-catenin. Cases without available slides and / or paraffin blocks were excluded from the study. The study was approved by the UFMG research ethics committee.

### Morphological evaluation

*In situ* lesions matching the LN morphological pattern were characterized according to the proliferation of generally small, dyshesive cells, with uniform round nuclei and clear cytoplasm. The acini are partially or completely expanded, but the lobular architecture is maintained [1]. Lesions classified as DCIS presented proliferation of monomorphic cells with regular distribution, and hyperchromatic nuclei forming regular secondary, rounded, and uniform lumens. The *in situ* lesions characterized as mixed pattern presented cytological or architectural features common to the ductal and lobular lesions and were classified into three main patterns according to criteria previously described by Jacobs *et al.*[3]: Group 1 – those presenting architectural and cytological findings of LN but with areas of comedo-type necrosis; Group 2 – those with CISM lesions characterized by small and uniform cells, either growing in a solid pattern with focal microacinar-like structures but with cellular dyshesion, or growing in a mosaic pattern with occasional intracytoplasmic vacuoles; Group 3 – those with marked cellular pleomorphism and nuclear atypia, however, the LN discohesive pattern remains.

### Immunohistochemical evaluation

Sequential 5 μm thick histological sections were obtained from the paraffin blocks from the breast specimens and mounted on silanized slides. Sections were deparaffinized in two consecutive baths of xylene, for 20 minutes each, and rehydrated in ethanol series with decreasing concentrations and finally in distilled water. For antigen retrieval, a buffer solution of 10 mM citrate pH 6.0 was used in an electrical pressure-cooker. Immunohistochemistry was performed automatically using Ventana Benchmark XT equipment (Ventana Medical Systems Inc., Tucson, AZ, USA). E-cadherin (clone 36) and β-catenin (clone 18) antibodies were used, at titers of 1:600 and 1:800, respectively. Antibodies were purchased from BD Transduction (cat# C19220), San Jose, CA, USA.

The non-biotinylated polymer system (Novolink^®^, Leica Microsystens) technique was used for reaction amplification. A diaminobenzidine (DAB) solution was used as chromogen and the slides were counter-stained using Harris hematoxylin. External positive and negative controls were used. Normal ducts and lobules, adjacent to the lesions and expressing E-cadherin and β-catenin in the epithelium, were used as internal controls.

**Figure 1:**
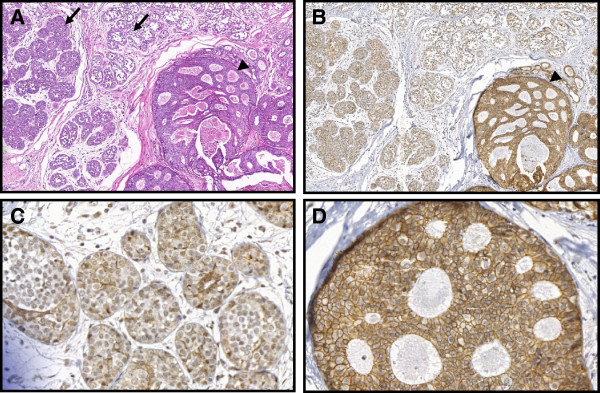
**Case # 1: Lobular neoplasia (arrows) and ductal carcinoma in situ of cribriform type (arrowheads) are present in the same breast field (hematoxylin and eosin; A – 100×).** Cells of ductal carcinoma stain positive (**B**,100× and **D**, 400×) and cells of lobular neoplasia are negative for E-cadherin (**B**, 100× and **C**, 400×).

Staining for E-cadherin and β-catenin was considered positive when the staining intensity around the entire circumference of the membrane was similar to that seen in the normal luminal epithelial cells. No staining was considered as negative (Figure 1).

## Results

Twenty-five cases of CISM were identified from the Breast Pathology Laboratory during the study period. The average patient age was 52.7 (± 11.5) years. Nineteen cases (76%) presented morphological pattern showing cytology and / or architectural mixed pattern (ductal and lobular), two cases (8%) showed lobular architectural pattern with nuclear pleomorphism, two cases (8%) showed mixed cytology and nuclear pleomorphism, and two cases (8%) showed comedonecrosis and nuclear pleomorphism (Table 1).

**Table 1:** **Morphology and immunophenotype of 25 breast carcinomas *in situ *** **with mixed pattern analyzed from breast biopsies**

	**Morphological pattern of *in situ *** **lesion**			**Morphology of *in situ *** **lesion with mixed pattern**			**E-cadherin immunophenotype**		**β-catenin immunophenotype**	
**Case #**	**Lobular**	**Ductal**	**Mixed**	**Nuclear pleomorphism**	**Comedonecrosis**	**Mixed cytology or architecture**	**Lobular areas**	**Ductal areas**	**Lobular areas**	**Ductal areas**
1	Yes	Yes	Yes	No	No	Yes	-	+	-	+
2	No	No	Yes	No	No	Yes	-	+	-	+
3	No	No	Yes	No	No	Yes	+	+	+	+
4	No	No	Yes	No	No	Yes	+	+	+	+
5	No	No	Yes	Yes	No	No	-	+	-	+
6	No	No	Yes	No	No	Yes	+	+	+	+
7	Yes	No	Yes	No	No	Yes	+	+	+	+
8	No	No	Yes	No	No	Yes	+	+	+	+
9	No	No	Yes	Yes	Yes	No	-	+	-	+
10	No	No	Yes	No	No	Yes	-	+	-	+
11	No	Yes	Yes	No	No	Yes	+	+	+	+
12	No	No	Yes	No	No	Yes	+	+	NA	NA
13	No	No	Yes	Yes	Yes	No	-	-	-	-
14	Yes	Yes	Yes	No	No	Yes	-	+	-	+
15	No	Yes	Yes	No	No	Yes	+	+	+	+
16	No	No	Yes	Yes	No	Yes	-	+	NA	NA
17	Yes	No	No	Yes	No	Yes	-	+	-	+
18	Yes	Yes	Yes	No	No	Yes	-	+	NA	NA
19	No	Yes	Yes	No	No	Yes	+	+	+	+
20	No	No	Yes	No	No	Yes	+	+	+	+
21	No	No	Yes	No	No	Yes	-	+	-	+
22	Yes	Yes	Yes	No	No	Yes	-	+	NA	NA
23	No	No	Yes	No	No	Yes	-	+	-	+
24	No	No	Yes	Yes	No	No	-	+	NA	NA
25	No	Yes	Yes	No	No	Yes	+	+	+	+

*NA* Not applied.

**Figure 2:**
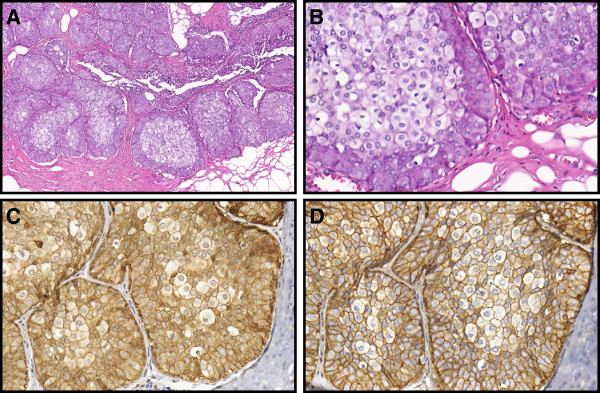
**Case # 8: Carcinoma in situ with mixed pattern (CISM) showing dual cell population (Group 2) stained for hematoxylin and eosin (A – 100×, B – 400×).** A stronger positive membrane staining for E-cadherin (**C** – 400×) and β-catenin (**D** – 400×) can be appreciated in the outer layer of cells of ductal pattern. A weaker staining is seen in cells of lobular pattern present in the center of the units.

Immunohistochemistry for E-cadherin was performed in all 25 cases. Eleven cases (44%) were positive for E-cadherin (Figure 2). Thirteen cases (52%) showed mixed immunophenotype with positive E-cadherin staining the ductal cells and negative in the lobular areas. In one case, the cells were completely negative for E-cadherin.

In all cases in which both markers were analyzed (20 cases) the immunohistochemical results agreed with both E-cadherin and β-catenin. Immunohistochemistry for β-catenin was not performed in five cases due to sample processing artifacts and insufficient material for the preparation of new slides.

**Figure 3:**
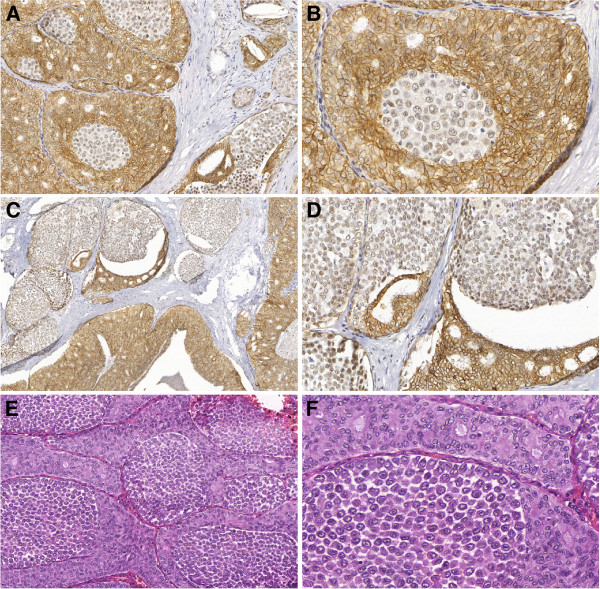
**Case # 16: Carcinoma in situ with mixed pattern (CISM) showing dual cell population stained for hematoxylin and eosin, involving a papilloma and adjacent ducts.** Areas of pleomorphic lobular pattern show discohesive atypical cells (arrows). Areas of “ductal” pattern show proliferation of cohesive cells with hyperchromatic nuclei forming secondary, rounded, and uniform lumens (arrowheads), (**A**, 200× and **B**, 400×). A mixed pattern resulted from a “collision” of both cell types in the same duct-lobular unit. “Ductal” cells are positive for E-cadherin (arrowheads) and lobular cells are negative (**C** and **E**– 200×, **D** and **F** – 400×).

Nineteen cases were composed by small, uniform cells varying from low to intermediate nuclear grade, growing in solid pattern, with some microacinar-like structures admixed with groups of low nuclear grade dyshesive cells, in a mosaic pattern. Of these, 11 (57.9%) presented positive immunohistochemistry for E-cadherin and β-catenin. In these cases, solid architecture with low-grade cytology was the most common morphological pattern. Eight of these cases (42.1%) presented the mixed immunophenotype and in four of them, the mixed pattern resulted from a “collision” of the lesions showing areas positive and areas negative for both markers in the same duct-lobular unit (Figure 3).

**Figure 4:**
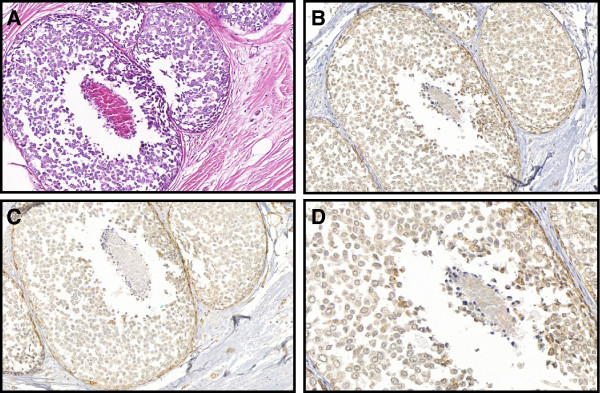
**Case # 13: Pleomorphic lobular carcinoma in situ showing discohesive atypical cells, with nuclear pleomorphism and comedonecrosis.** Hematoxylin and eosin (**A** – 100×). Lobular cells are negative for E-cadherin (**B** – 100×; **D** – 400×) and for β-catenin (**C** – 100×).

Two cases presented the lobular architectural pattern with nuclear pleomorphism and two cases presented mixed cytology and nuclear pleomorphism. The last two cases were considered as mixed immunophenotype. Of the two cases that presented comedonecrosis and nuclear pleomorphism, one was completely negative for both markers (Figure 4) and the other presented cells positive and cells negative for both markers (Table 2).

**Table 2:** **Category morphology and immunophenotype for E-cadherin of carcinoma *in situ *** **with mixed pattern**

**Morphology**	**Cases**	**E-cadherin**		
		**Positive**	**Negative**	**Mixed**
**Group 1**	0	0	0	0
**Group 2**	19	11	0	8
**Group 3**	2	0	0	2
**Group 2 and 3**	2	0	0	2
**Group 1 and 3**	2	0	1	0
**Total**	25	11	1	13

## Discussion

In this study, we sought to evaluate the expression of E-cadherin and β-catenin for the immunophenotypical characterization of CISM. We also searched for potential morphological patterns that could help in the diagnosis of different types of CISM lesions. We adopted the morphological classification described by Jacobs *et al.* that defines CISM as “carcinomas with indeterminate features”[3]. According to this classification CISM lesions are divided in three main groups, namely: (I) presence of necrosis, (II) cytology and / or mixed architecture, and (III) nuclear polymorphism. This classification is highly reproducible and addresses the main morphological groups described in our study.

A total of 25 CISM cases were evaluated in this study. The most common morphological pattern of lesions identified belonged to group (II) with 19 out of 25 cases (76%), followed by group (III) (2/25 cases - 8%), and overlapping patterns between groups (I) and (II) (2/25 cases - 8%) and groups (II) and (III) (2/25 cases - 8%). Our findings are in agreement with those reported by Jacobs *et al.* who observed, in 28 cases of CISM, 60% of the lesions in group (II) (17/28 cases), 21% in group (I) (5/28 cases) , and 18% in group (III) (5/28 cases).

However, it is noteworthy that the terminology and morphologic criteria used for the diagnosis of CISM are heterogeneous. Fisher *et al.* termed it as “ductolobular carcinoma *in situ*” lesions with monomorphic cells with foci of necrosis or cribriform pattern [4]. Acs *et al.* described 14 cases of CISM referring to the lesions as “with ductal carcinoma *in situ* and lobular features” and adopted, as a diagnostic criteria, LN *in situ* lesions with cytological and architectural patterns, with central areas of comedonecrosis or lobules, or large duct units populated by non-cohesive cells with marked nuclear pleomorphism [19]. Maluf *et al.* analyzed 12 cases of “solid low grade carcinoma *in situ* of the breast” and included “low-grade solid DCIS, LCIS and DCIS and LCIS associated with invasive carcinomas of any type. Cases showing only unequivocal areas of LCIS or DCIS of nonsolid type were excluded” [20].

Even among experts in the pathology of breast tumors, the descriptions of these lesions are divergent. Page and Anderson state that in most cases an attempt should be made to classify the lesions as LN or DCIS [21]. However, on rare occasions this might not be possible and the diagnosis of “*in situ* carcinoma of ductal or lobular type” needs to be made. These authors recommend that if more than one focus of necrosis is found, the lesion should not be classified as LN. Rosen describes two main types of CISM: “concurrent intraductal and lobular carcinoma *in situ*” for lesions that present a cytology of lobular pattern and distended ducts and central necrosis or calcifications, and “coexistent intraductal and lobular carcinoma *in situ* in a single duct-lobular unit.” The author uses this description to refer to the more unusual intraductal lesions characterized by the presence of two distinct architectural and cytological patterns [22].

Recently, *in situ* lesions with lobular cytological features of classic LCIS but with marked nuclear pleomorphism, comedonecrosis, and with or without apocrine cytology have been described as pleomorphic LCIS [1]. Some reports suggest that these variants are more aggressive than classic LN and a surgical treatment similar to that applied to DCIS is recommended. However , there are no prospective epidemiological studies showing that these variants have different clinical significance and appropriate management of pleomorphic LCIS is currently uncertain [1].

In our series, we observed a frequent association between immunophenotype and morphology (cytoarchitectural features). Lesions in group (II), with solid architecture and low-grade cytology, were more often associated with expression of E-cadherin. Our data differ from those reported by Acs *et al.*, in which no expression of E-cadherin was observed in the 14 CISM cases analyzed. The most frequent morphological pattern observed in that study was presence of lobular cytology with comedonecrosis (n = 9 ) [19]. Maluf *et al.* analyzed 12 CISM cases and detected E-cadherin expression in five, while another five cases showed no expression of the protein and two presented a mixed population of cells in this regard. These authors did not observe the prevalence of a specific morphological pattern over others [20]. Similarly to the study by Jacobs *et al.*, lesions in group (II) were the most frequent lesion associated with expression of E-cadherin in our study, however we observed them at a higher frequency. A dual cell population in the same terminal duct-lobular unit was observed in four cases. This is likely due to the coexistence of LN and DCIS in the same terminal duct-lobular unit.

Since first reported, the immunohistochemical reaction of E-cadherin has been proposed as an aiding tool in the differential diagnosis between ductal and lobular lesions, either invasive or *in situ*. However, it should be noted that up to 15% of lobular lesions may exhibit aberrant expression of E-cadherin and thus, the lack of E-cadherin expression should not be used as the sole criterion for LN diagnosis [12]. Choi *et al.* observed variability in the immunohistochemical staining of E-cadherin, and detected abnormal staining patterns, both in ductal and lobular lesions, making the differential diagnosis between *in situ* lobular and invasive lesions very difficult through immunohistochemistry [10]. An alternative to reduce this interference and improve diagnosis is the combined use of immunohistochemical markers of the catenin pathway. Using IHC and molecular biology techniques Da Silva *et al.* analyzed three cases presenting morphological characteristics and genotyping that agreed with invasive lobular carcinoma, with nonetheless aberrant expression of E-cadherin. Of these three cases, two did not express β-catenin, indicating that the formation of the cadherin-catenin complex, which is required for the normal function of the cell and maintenance of tissue architecture, including cell adhesion, failed [12,23]. In our study, we observed that expression of E-cadherin agreed with expression of β-catenin in all cases here observed.

Other explanations for the abnormal expression of E-cadherin found in other studies may be related to technical difficulties and pitfalls that may occur during the different stages in the immunohistochemical reaction. In our study, we had some difficulties in the pre-analytical reaction such as material loss and weak staining in some cases. This may reflect the fact that we used specimens coming from the routine diagnosis laboratory of a general hospital; and other cases were sent to us for a second opinion. In many cases, there was no control of the pre-analytical phase or standardization of time of formalin fixation and unbuffered formalin was used. Goldstein *et al.* showed that the reactivity level varies with the number of blocks and thickness of the sample sections in the pre-analytical process [2]. Different clones of antibodies against E-cadherin and different antigens may also have an effect on the quality of the immunohistochemical staining. A comparison between two types of antibodies revealed discrepancy in the staining of lobular lesions in 6.4% of the cases [7]. Finally, there is a lack of consensus regarding the interpretation of the positivity of immunohistochemical staining of E-cadherin. The established cutoff of a positive signal varies between basal membrane expression and presence of any positivity to 20% of expressing cells [12,23]. Semi-quantitative evaluations of the intensity of staining and association of different criteria forming scores of staining intensity have also been proposed [19].

Other immunohistochemical markers have been suggested to aid in the diagnosis of CISM. The p120 catenin is an intracellular protein that promotes the binding between the complex of catenins and cell cytoskeleton. When E-cadherin expression is absent, p120 catenin is dispersed in the cytoplasm, which explains its expression in the cytoplasm in LN, and in the membrane in DCIS [23,24].

## Conclusions

The immunophenotypic characterization of carcinomas *in situ* using E-cadherin and β-catenin, combined with the analysis of cytological and architectural patterns, is a useful tool for the morphological and immunophenotypical classification of CISM. However, a negative staining for these markers should not be used as the sole criterion of lobular phenotype because aberrant expression in lobular neoplasia and loss of expression in ductal cancers can both occur.

## Abbreviations

ALH: Atypical lobular hyperplasia; CISM: Carcinoma *In Situ* with mixed pattern; DCIS: Ductal carcinoma *In Situ*; LN: Lobular neoplasia; LCIS: Lobular carcinoma *In Situ*; IHC: Immunohistochemistry; E-cadherin: Human epithelial cadherin; PLCIS: Pleomorphic lobular carcinoma *In Situ*; UFMG: Federal University of Minas Gerais.

## Competing interests

The authors declare that they have no competing interests.

## Authors’ contributions

*DSG* conceived the study, participated in the histological review, and drafted the manuscript. *SSP* participated in the design of the study. RMG: Performed the immunohistochemistry reactions. *HG* participated in design and coordination of the study, participated in the histological review, and drafted and reviewed the manuscript. All authors have read and approved the final manuscript.

## References

[B1] Lakhani SR Ellis IO Schnitt SJ Tan PH van de Vijver MJ World Health Organization WHO classification of tumours of the breast 2012 4 Lyon: IARC

[B2] Goldstein NS Bassi D Watts JC Layfield LJ Yaziji H Gown AM E-cadherin reactivity of 95 noninvasive ductal and lobular lesions of the breast. Implications for the interpretation of problematic lesions Am J Clin Pathol 2001 115 534 542 11293901 10.1309/B0DD-4M7H-GJG1-7KCW

[B3] Jacobs TW Pliss N Kouria G Schnitt SJ Carcinomas in situ of the breast with indeterminate features: role of E-cadherin staining in categorization Am J Surg Pathol 2001 25 229 236 11176072 10.1097/00000478-200102000-00011

[B4] Fisher B Costantino JP Wickerham DL Redmond CK Kavanah M Cronin WM Vogel V Robidoux A Dimitrov N Atkins J Tamoxifen for prevention of breast cancer: report of the national surgical adjuvant breast and bowel project P-1 study J Natl Cancer Inst 1998 90 1371 1388 9747868 10.1093/jnci/90.18.1371

[B5] Schnitt SJ Morrow M Lobular carcinoma in situ: current concepts and controversies Semin Diagn Pathol 1999 16 209 223 10490198

[B6] Lakhani SR Audretsch W Cleton-Jensen AM Cutuli B Ellis I Eusebi V Greco M Houslton RS Kuhl CK Kurtz J The management of lobular carcinoma in situ (LCIS). Is LCIS the same as ductal carcinoma in situ (DCIS)? Eur J Cancer 2006 42 2205 2211 16876991 10.1016/j.ejca.2006.03.019

[B7] Liberman L Clinical management issues in percutaneous core breast biopsy Radiol Clin North Am 2000 38 791 807 10943278 10.1016/s0033-8389(05)70201-3

[B8] Nagi CS O’Donnell JE Tismenetsky M Bleiweiss IJ Jaffer SM Lobular neoplasia on core needle biopsy does not require excision Cancer 2008 112 2152 2158 18348299 10.1002/cncr.23415

[B9] Bratthauer GL Moinfar F Stamatakos MD Mezzetti TP Shekitka KM Man YG Tavassoli FA Combined E-cadherin and high molecular weight cytokeratin immunoprofile differentiates lobular, ductal, and hybrid mammary intraepithelial neoplasias Hum Pathol 2002 33 620 627 12152161 10.1053/hupa.2002.124789

[B10] Choi YJ Pinto MM Hao L Riba AK Interobserver variability and aberrant E-cadherin immunostaining of lobular neoplasia and infiltrating lobular carcinoma Mod Pathol 2008 21 1224 1237 18587329 10.1038/modpathol.2008.106

[B11] Gomes DS Balabram D Porto SS Gobbi H Lobular neoplasia: frequency and association with other breast lesions Diagn Pathol 2011 6 74 21827679 10.1186/1746-1596-6-74 PMC3170574

[B12] Da Silva L Parry S Reid L Keith P Waddell N Kossai M Clarke C Lakhani SR Simpson PT Aberrant expression of E-cadherin in lobular carcinomas of the breast Am J Surg Pathol 2008 32 773 783 18379416 10.1097/PAS.0b013e318158d6c5

[B13] Goldstein NS Kestin LL Vicini FA Clinicopathologic implications of E-cadherin reactivity in patients with lobular carcinoma in situ of the breast Cancer 2001 92 738 747 11550142

[B14] Baranwal S Alahari SK Molecular mechanisms controlling E-cadherin expression in breast cancer Biochem Biophys Res Commun 2009 384 6 11 19379710 10.1016/j.bbrc.2009.04.051 PMC2700729

[B15] Andrews JL Kim AC Hens JR The role and function of cadherins in the mammary gland Breast cancer res 2012 14 203 22315958 10.1186/bcr3065 PMC3496113

[B16] Wangefjord S Brandstedt J Lindquist KE Nodin B Jirstrom K Eberhard J Associations of beta-catenin alterations and MSI screening status with expression of key cell cycle regulating proteins and survival from colorectal cancer Diagn Pathol 2013 8 10 23337059 10.1186/1746-1596-8-10 PMC3599130

[B17] Cheng H Liang H Qin Y Liu Y Nuclear beta-catenin overexpression in metastatic sentinel lymph node is associated with synchronous liver metastasis in colorectal cancer Diagn Pathol 2011 6 109 22053859 10.1186/1746-1596-6-109 PMC3222611

[B18] Tsanou E Peschos D Batistatou A Charalabopoulos A Charalabopoulos K The E-cadherin adhesion molecule and colorectal cancer. A global literature approach Anticancer Res 2008 28 3815 3826 19189669

[B19] Acs G Lawton TJ Rebbeck TR LiVolsi VA Zhang PJ Differential expression of E-cadherin in lobular and ductal neoplasms of the breast and its biologic and diagnostic implications Am J Clin Pathol 2001 115 85 98 11190811 10.1309/FDHX-L92R-BATQ-2GE0

[B20] Maluf HM Swanson PE Koerner FC Solid low-grade in situ carcinoma of the breast: role of associated lesions and E-cadherin in differential diagnosis Am J Surg Pathol 2001 25 237 244 11176073 10.1097/00000478-200102000-00012

[B21] Page DL Anderson TJ Diagnostic histopathology of the breast 1987 Edinburgh; New York: Churchill Livingstone

[B22] Rosen PP Rosen’s breast pathology 2008 3 Philadelphia: Lippincott Williams & Wilkins

[B23] Dabbs DJ Bhargava R Chivukula M Lobular versus ductal breast neoplasms: the diagnostic utility of p120 catenin Am J Surg Pathol 2007 31 427 437 17325485 10.1097/01.pas.0000213386.63160.3f

[B24] Sarrio D Perez-Mies B Hardisson D Moreno-Bueno G Suarez A Cano A Martin-Perez J Gamallo C Palacios J Cytoplasmic localization of p120ctn and E-cadherin loss characterize lobular breast carcinoma from preinvasive to metastatic lesions Oncogene 2004 23 3272 3283 15077190 10.1038/sj.onc.1207439

